# Mood and cognition in healthy older European adults: the Zenith study

**DOI:** 10.1186/2050-7283-2-11

**Published:** 2014-05-02

**Authors:** Ellen EA Simpson, Elizabeth A Maylor, Christopher McConville, Barbara Stewart-Knox, Natalie Meunier, Maud Andriollo-Sanchez, Angela Polito, Federica Intorre, Jacqueline M McCormack, Charles Coudray

**Affiliations:** Psychology Research Institute, University of Ulster, Londonderry, UK; Northern Ireland Centre for Food and Health (NICHE), University of Ulster, Coleraine, Northern Ireland UK; Department of Psychology, University of Warwick, Coventry, UK; Division of Psychology, University of Bradford, Yorkshire, UK; CHU Clermont Ferrand, Unité d’Exploration en Nutrition, CRNH Auvergne, Clermont-Ferrand, France; Université Joseph Fourier, Saint-Martin-d’Hères, France; Agricultural Research Council–Research Centre on Food and Nutrition (CRA-NUT), Rome, Italy; UMR 866 (Dynamique Musculaire & Métabolisme) INRA, Place Viala, Montpellier, France; School of Psychology, University of Ulster, Cromore Road, BT521SA Coleraine, County Londonderry Northern Ireland

**Keywords:** Mood, Affect, PANAS, Cognition, CANTAB, Older adults

## Abstract

**Background:**

The study aim was to determine if state and trait intra-individual measures of everyday affect predict cognitive functioning in healthy older community dwelling European adults (n = 387), aged 55-87 years.

**Methods:**

Participants were recruited from centres in France, Italy and Northern Ireland. Trait level and variability in positive and negative affect (PA and NA) were assessed using self-administered PANAS scales, four times a day for four days. State mood was assessed by one PANAS scale prior to assessment of recognition memory, spatial working memory, reaction time and sustained attention using the CANTAB computerized test battery.

**Results:**

A series of hierarchical regression analyses were carried out, one for each measure of cognitive function as the dependent variable, and socio-demographic variables (age, sex and social class), state and trait mood measures as the predictors. State PA and NA were both predictive of spatial working memory prior to looking at the contribution of trait mood. Trait PA and its variability were predictive of sustained attention. In the final step of the regression analyses, trait PA variability predicted greater sustained attention, whereas state NA predicted fewer spatial working memory errors, accounting for a very small percentage of the variance (1-2%) in the respective tests.

**Conclusion:**

Moods, by and large, have a small transient effect on cognition in this older sample.

## Background

With increased longevity and changing population demographics there is a need to understand factors that promote and maintain healthy agein`g (Eurostat, 2012), which is characterised by enhanced cognitive and emotional functioning in some older populations (Depp and Jeste, 2009; Paulson et al. 2011; Rowe and Kahn 1997). There is a renewed interest in what happens to everyday mood with age and the implications of this for health and well-being (Ready et al. 2011). Changes in mood, induced in laboratory settings, have been reported to influence cognitive performance in different age groups, such as undergraduate students (Oaksford et al. 1996; Phillips et al. 2002), younger males (Roiser et al. 2007), younger adults (Chepenik et al. 2007) and older adults (Kensinger et al. 2007; Phillips et al. 2002). Further research is required to gain a better understanding of the relationship between everyday mood (affect) and cognitive performance in older adults.

Cognitive function refers to the underlying processes involved in attention, perception, memory and learning (Eysenck, 2006). Most of the cross-sectional research on healthy community dwelling older adults suggests that attention, working memory and speed of information processing decline gradually in adults from their 20s up to 60 years of age (Craik and Byrd, 1982; Kramer et al. 2004; Salthouse, 2009), with a more rapid decline beyond 70 years of age (Ronnlund et al. 2005; Schaie, 2005), even when investigated longitudinally (Salthouse, 2010). Other aspects of memory such as vocabulary and general knowledge do not appear to change up to 60 years of age (Salthouse, 2009). There is also some evidence for sex differences in working memory and reaction times (DeLuca et al. 2003; Meinz and Salthouse, 1998), with men having better cognitive performance on these tests than women. Additionally, higher socio-economic status has been associated with better cognitive function in later life (Herrmann and Guadagna, 1997; Richardson, 1999) and will be investigated as a potential predictor of memory in the current study.

Mood or affect is a subjective state conceptualized as two independent continua, positive affect (PA) and negative affect (NA) (Watson et al. 1988; Watson and Tellegen, 1985). PA reflects states such as joy, alertness, and enthusiasm, while NA measures the amount of unpleasantness or dissatisfaction the person is experiencing (Watson and Clark, 1992). Longitudinal and cross-sectional research has suggested that PA remains relatively stable across the lifespan (Charles et al. 2001), with some studies showing a slight increase in PA with age (Mroczek and Kolarz, 1998). NA, in contrast, decreases throughout life until around 60 years of age where the decline becomes less marked (Carstensen et al. 2000; Charles et al. 2001; Mroczek and Kolarz, 1998). Other studies report no change in affect with age (Smith and Baltes 1993; Vaux and Meddin, 1987). These mixed findings may be explained by differences related to socio-demographic factors, such as sex and social class, and may also influence affect in later life (Watson and Clark, 1999), both of which will be investigated in the current study.

Existing research has focused on the two separate constructs of PA and NA, and has tended to induce changes in affect artificially in the laboratory context (Oaksford et al. 1996; Phillips et al. 2002). However, in everyday life affect does not remain static but changes in response to the environment (Watson, 2000). It has been suggested that affect variability is related to mood disorders (Eastwood et al. 1985) and psychological vulnerability in community dwelling adults (Murray et al. 2002). It has been proposed that the underlying mechanisms responsible for trait affect may differ from those regulating or stabilising momentary affect and that the two should be investigated separately (Cowdry et al. 1991). There is very little research which has examined this in healthy ageing. There is a need to better understand the relationship between fluctuations in affect and cognitive function in everyday life and particularly in older adults who are at risk of impaired cognitive function. This will be investigated in the current study by examining variability in affect.

Early studies of affect and information processing began in the 1980’s (Bless and Fiedler, 2006). These early studies tended to focus on differences in processing while experiencing PA and NA. PA was associated with less stringent processing of information and making quick decisions; NA involved more systematic and vigilant processing of information (Clark and Isen, 1982; Schwarz, 1990). These findings were explained by differences in motivation during PA and NA; the former would result in the participant wishing to maintain the PA for as long as possible, so not engaging in stressful processing. With NA, participants are more likely to engage in systematic processing to alleviate the negative state (Clark and Isen, 1982; Schwarz, 1990).

A number of theories have been put forward to explain the potential mechanisms underlying the relationship between affect and cognition. Some studies have suggested that affect may exert an effect on cognitive function by influencing motivational and attention processes (Clore and Starbeck, 2006; Forstmeier and Maercker, 2008; Hess et al. 2012). Cognitive load theory suggests that heightened affect, both positive and negative, may overload cognitive resources, producing a lack of focus, reduced concentration and impaired performance, by limiting the “cognitive control” over the processes needed to complete a task (Brose et al. 2012; De Pisapia et al. 2008), especially tasks requiring effort such as executive function (Martin and Kerns, 2011; Matthews and Campbell, 2011; Phillips et al. 2002) and episodic memory tasks (Allen et al. 2005). Some fMRI research suggests that affect is related to hemispheric asymmetry in the prefrontal cortex in response to specific task related activation, with PA associated with RH activation and NA with LH activation. It has been suggested that this may be related to cognitive load and competition for brain processes required for cognition and affect.

Capacity limitation theory (Siebert and Ellis, 1991) suggests that both PA and NA overload cognitive resources due to increased intrusive thoughts and reduce the capacity of working memory. Changes in NA may lead to attempts to regulate affect and reduced resources and reduced cognitive performance. Some evidence to support this has been found in a study of younger adults (Riediger et al. 2011). Other researchers argue that PA leads to more flexibility which facilitates problem solving and greater innovation (Isen, 1999). Mitchell and Phillips (2007) concluded in their review that PA reduces executive function, such as planning and working memory.

Previous research looking at the influence of affect on cognitive function has relied heavily on artificial laboratory based affect induction methods and has focused upon younger adults (Chepenik et al. 2007; Oaksford et al. 1996; Roiser et al. 2007). Of the few studies on older adults, some have compared them to younger groups (Kensinger et al. 2007; Phillips et al. 2002), or have focused on PA (Hill et al. 2005) or NA (Rabbitt et al. 2008) only, with conflicting results. Few studies have looked at naturally occurring affect and how these relate to cognitive functioning in healthy older individuals, even though induced affect and natural affective states may influence cognitive function in different ways (Parrott and Sabini, 1990). Some people’s affect varies widely across time whilst others remain stable (McConville and Cooper, 1997), but few studies have considered this with respect to ageing. Röcke et al. (2009) compared everyday affect and affect variability in young (20-30 years) and older (70-80 years) adults and found little difference between the groups in relation to mean PA and NA and they observed less variability in PA and NA in the older age group. In a more recent study, comparing affect variability in young and old participants, similar results were reported but it was suggested that more research is needed to fully understand age-related differences in affect variability (Brose et al. 2013).

Whereas PA has a well-established circadian rhythm and tends to vary constantly in response to the environment, NA tends to be more stable unless faced with a stressor or major life event (Watson, 2000). Some recent studies looking at natural affect in younger adults reported that increases in NA (assessed retrospectively) produced a detrimental effect on working memory and attention (Aoki et al. 2011; Brose et al. 2012). The detrimental effect of NA may occur during information processing in working memory (Li et al. 2010). The current study will examine fluctuation in everyday affect and whether this is related to cognitive performance in older people.

In the current study, mood was assessed by repeated measures over four consecutive days at designated times throughout the day. Commonly, mean levels of PA and NA are computed from each person’s repeated affect assessments. These measures reflect an individual’s levels of trait PA and NA. To assess the extent to which affect fluctuates, the standard deviation is computed for each person’s PA and NA scores from repeated assessments. The standard deviation provides a measure of affect variability (Murray et al. 2002), which has distinct characteristics of a trait, largely independent of affect levels (McConville and Cooper, 1999; Murray et al. 2002) and is an important individual difference construct in the study of affect.

A better understanding of emotional regulation in older adults is required and particularly the shift to higher PA, reported in some studies of older individuals (Depp et al. 2010; Mather and Carstensen, 2005). A better understanding of the interaction between socio-demographic variables and affect and how these relate to cognitive function in later life is also required. Thus the aim of the current study was to determine if state, trait and affect variability measures of everyday affect predict cognitive function in healthy older adults aged 55+ years, after controlling for socio-demographic (age, sex and social class) variables, which may mediate any relationship between cognition and affect (Santos et al. 2013).

## Method

This study was conducted in accordance with the declaration of Helsinki and was approved by the ethics committees within each centre: the University of Ulster’s Research Ethics committee, UK; Advisory Committee on the Protection of Persons in Biomedical Research Clermont Ferrand, France; Ethics committee of the Centre Hospitalier Universitaire de Grenoble, France and Ethical Committee of the Italian National Research Centre on Aging (I.N.R.C.A.), Rome.

### Participants

Volunteers were recruited through community groups and organisations serving older adults as part of the Zenith Study. Centres in Rome, Italy and Grenoble, France recruited adults aged 70-87 years and Northern Ireland, UK and Clermont-Ferrand, France recruited adults aged 55-70 years. Exclusion criteria were adapted from the SENIEUR protocol (Ligthart et al. 1984) for demographic suitability. Effort was made to recruit equal numbers of males and females. All volunteers were required to give full, informed written consent prior to taking part in the research.

At screening, a medical examination was given which included liver and kidney function tests, full blood and lipid profiles, blood pressure, heart rate, anthropometric measurements, assessment of dietary habits, consumption of tobacco (with an inclusion criterion of <10 cigarettes per day) and alcohol (which had to be within the recommended amount of less than 30 g and 20 g of alcohol per day, respectively, for males and females) (Polito et al. 2005). Volunteers were screened for depression by means of the 15-item Geriatric Depression Scale (Yesavage et al. 1983) and were excluded if they scored 5 or more. The Mini Mental State Examination (Folstein et al. 1975) was used to screen for dementia, and participants were excluded if they scored less than 24. Socio-demographic information was obtained using a self-report questionnaire derived from the EPIC study and described elsewhere (Simpson et al. 2005).

### Cognitive measures

Cognitive function was assessed using the Cambridge Automated Neuropsychological Test Battery (CANTAB; Morris et al. 1986). CANTAB has proven brain-to-behaviour reliability (Luciano and Nelson, 2002; Robbins et al. 1997) and test-retest reliability (Louis et al. 1999). Evidence of construct validity was obtained from studies of neurological patients with disorders that affect specific areas of the brain (Owen et al. 1996; Owen et al. 1996), patients with psychiatric disorders (Elliott and Sahakian, 1995) and neuroimaging studies (Coull et al. 1996). CANTAB has been deemed suitable for use with older adults (Robbins et al. 1994).

A detailed account of the cognitive tests used in this research can be found in Maylor et al. (2006). The tests used in the current study are sensitive to changes in cognition with age and neurodegeneration. Pattern recognition memory (PRM) is a two alternative forced-choice test of visual recognition memory which required participants to memorise and recall abstract patterns. The dependent variable was mean latency in milliseconds (ms) which reflects the mean length of time taken to select the correct pattern. This test activates the temporal lobe, hippocampus and amygdala regions of the brain (Robbins et al. 1997). A test of Spatial Working Memory (SWM) required participants to search through a number of boxes, presented on the screen, to locate blue tokens, which were then used to fill up a column on the right hand side of the screen. Only one token was hidden at a time and once a token was found no further tokens were hidden in that box for the duration of the trial. The trials increased in difficulty. The outcome measure was the total number of search errors made over all of the trials. This test activates the temporal and frontal lobe regions of the brain (Robbins et al. 1997). A 5-choice reaction time task (5CRT) assessed the participant’s speed of responding to a visual stimulus that appeared on the screen. The dependent measure (correct trials only) was the mean latency (time taken) in milliseconds from the appearance of the stimulus to the release of the press-pad (i.e., reaction time). Match to sample visual search (MTS) is a pattern matching task assessing sustained attention, for which the dependent variable was mean correct latency (in milliseconds). The latter two tests are measures of attention and activate the fronto-striatal circuitry (Robbins et al. 1997). All of these tests activate areas of the brain sensitive to ageing and also areas thought to be involved in the interaction between affect and cognitive function (e.g. see Forgas, 2008; Mitchell and Phillips, 2007).

### Affect measures

The Positive and Negative Affect Schedule (PANAS) (Watson et al. 1988) is a 20-item scale with 10 items assessing PA (happy, alert) and 10 items measuring NA (nervous, irritable). These are considered higher level affective states and account for most of the important variance from many discrete affects (Cooper and McConville, 1989). Momentary affect was measured four times a day for four consecutive days: upon rising; at 14.30; after dinner (17.00-18.00) and at 22.30. Participants were asked to complete the questionnaire based on how they felt at that particular moment when giving their answers. Responses were recorded on a five-point Likert scale ranging from “not at all” = 1, to “extremely” = 5. A score for each scale was obtained by summing item scores. The scales have high internal consistency, with Cronbach’s alpha ranging from .84 to .90 for the PA scale and .84 to .87 for the NA scale (Watson and Walker, 1996). The scales have convergent, construct and discriminant validity and have been previously employed in studies of older adults (Segal, Bogaards, Becker, and Chatman, 1999; Watson et al. 1988).

Trait affect was assessed by 16 momentary scores for PA and 16 momentary scores for NA. The dependent trait measures of affect were based on overall intra-individual means and SDs for PA and NA for the four days; this method has been used successfully in other studies (Duffy et al. 2006; McConville and Cooper, 1997; Williams et al. 2006). Each person’s mean provides a summary of their affective states over the four days, while the SD gives an indication of the extent to which their PA and NA scores fluctuated over the four days. State affect was assessed by a single PANAS scale completed prior to the CANTAB tests.

### Procedure

Each centre adopted the same protocol for gathering mood data and conducting the cognitive tests as follows. Following successful screening and ten days prior to assessment at the research centre, participants received an information pack that included full written instructions as to how to record their affect. Each pack contained an A5 size PANAS booklet, with 16 PANAS questionnaires, 4 to be completed per day for 4 days, which were labelled day 1 to day 4, with the designated times for completion written on them. Diaries were supplied to record any difficulties encountered with the protocol. On the second day of recording affect, participants were contacted by phone and any problems were addressed. Participants returned their completed PANAS scales to the research centre at the end of the four days, which corresponded with their next research appointment.

During this visit, participants attended the centre early in the morning and were given breakfast (cereal, fruit juice, toast, and decaffeinated tea or coffee). Following breakfast, they completed one single PANAS and then undertook the cognitive tests, presented in the following order: a motor screening test, pattern recognition memory, spatial span (results not presented), spatial working memory, simple reaction time (results not presented), 5-choice reaction time, and match to sample (visual search). Participants were seated approximately 0.5 m from the screen. All instructions for tests were given verbatim from the CANTAB manual by a trained researcher. Completion of testing took 35-40 minutes, after which participants were thanked for taking part in the study.

### Data analyses

A series of 2 (age group: 55-70 yrs vs. 70-87 yrs) * 2 (sex) and 2 (age group: 55-70 yrs vs. 70-87 yrs) * 3 (social class: professional, skilled and unskilled) ANOVAs were conducted to establish age, sex and social class differences and interaction effects for measures of state, trait affect, affect variability and cognition. Pearson bivariate correlations were carried out to look at the initial relationships between measures of cognitive function and measures of state and trait PA, NA and variability. In order to determine what predicted cognitive function, a series of hierarchical regression analyses were carried out. Separate analyses were carried out for PA and NA (state, trait and variability measures), one for each of the cognitive measures (PRM, SWM, 5CRTand MTS). Dummy variables were created for category variables sex and social class. The first step in the regression analyses involved entering socio-demographic information of age in years, sex and social class, followed by state affect (either PA or NA) in step two, and lastly, in step three, trait measures of PA and NA and their variability were entered. It should be noted that each step, after step one, included the variable(s) from the previous step(s).

Prior to data analyses, the data were checked for normality by first examining statistics for skewness and kurtosis. Generally, the closer to 0 both of these statistics are the more likely the sample scores are normally distributed. There are rules of thumb however which indicate that skewness of a range of +2 to -2 does not require data transformation (Kline, 2010; Tabachnick and Fiddell, 2007) and for kurtosis anything over 10 should be transformed (Kline, 2010). Based on this guide, and examination of normal distribution graphs (Tabacknick and Fiddell, 2007), it was decided to transform PRM scores (high skew and high kurtosis) and trait NA means (high skew), together with the remaining NA measures, using a logarithmic (base 10) transformation. In the case of NA variability, a number of zero scores were recorded for this variable and in order to conduct the transformation a constant was added to all scores (in this case 1) prior to transformation. Note that the results were not qualitatively affected by these transformations.

Checks on multicollinearity were performed for all hierarchical regression analyses using the variance inflation factor (VIF) statistic by which acceptable values must not exceed 10 (Tabachnick and Fiddell, 2007). In the current analyses, all VIF values were within the recommended range. This was supported by the Pearson’s bivariate correlation matrix for affect and cognitive function, which indicated that the majority of correlations were relatively small. With the exception of trait NA mean and *SD* (r = .74), and state and trait PA (r = .72), VIF was low for all predictors. For this reason state and trait PA and NA measures entered separately. Outliers and their influence (as assessed by leverage, Cook’s Distance), normality, linearity, homoscedasticity and independence of residuals were checked by reviewing probability plots and scatterplots of the regression standardized residuals and were deemed acceptable.

The software program G*Power 3 (Faul et al. 2007) was used to conduct a power analysis. This indicated that with seven predictors in the final step, an alpha of .01, and a small to medium effect size f^2^ = .087 (corresponding to an R^2^ = .08), a sample size of 259 would result in a power value greater than .95. In our study the sample size was greater than this.

## Results

### Socio-demographic information

Approximately 10-15% of those initially approached contacted the research group and volunteered to take part in the study. All of the participants were apparently healthy community dwelling adults aged 55-87 years (N = 387). Socio-demographic information for the sample is given in Table 1. Almost equal numbers of males and females were recruited within each age group (*X*^2^ = 0.29, *df* = 3, *p* = 0.962). The groups were comparable with regards to occupational categories with the exception of the older group who had a slightly lower percentage of professional occupations (*X*^2^ = 24.04, *df* = 6, *p* = 0.001).

**Table 1:** **Socio-demographic variables of age, sex and social class for each age group**

	Age groups	
Variable	55-70 yrs	70-87 yrs
**N**	188	199
**Mean (SD) age in years**	61.8 (4.4)	74.3 (3.7)
**Sex (%)**		
**Male**	49.5	51.8
**Female**	50.5	48.2
**Social class (%)**		
**Professional**	43.2	33.2
**Skilled**	48.1	55.8
**Semi-unskilled**	8.7	11.1

### Sex, age group and social class differences in state and trait affect measures and cognitive function

A number of sex and age differences emerged for affect and cognition (see Table 2). Females reported slightly lower levels of trait PA and marginally higher trait NA than males and showed greater variability in these measures over time. Older adults (70-87 yrs) showed lower levels of state and trait PA and less variability in PA than the younger group (55-70 yrs); trait NA was slightly higher in the older age group.

**Table 2:** Means (and SDs) for the state and trait measures of everyday mood and cognitive variables, also showing sex and age differences and sex*age interactions

Variable	55-70 yrs			70-87 yrs					
	Males	Females	Group mean	Males	Females	Group mean	Sex differences	Age differences	Sex * Age interactions
**State mood**									
**State PA**	34.10 (5.9)	32.85 (6.0)	33.48 (5.9)	28.22 (9.2)	27.35 (9.5)	27.81 (9.3)	*p* = 0.201	***p*** **< 0.001**	*p* = 0.821
**State NA**	13.72 (4.3)	14.38 (5.7)	14.05 (5.0)	13.78 (5.5)	13.96 (5.6)	13.87 (5.6)	*p* = 0.454	*p* = 0.753	*p* = 0.669
**Trait mood and mood variability measures**									
**Trait PA**	28.63 (5.4)	26.39 (5.2)	27.52 (5.4)	22.85 (7.8)	22.14 (7.4)	22.52 (7.6)	***p*** **= 0.034**	***p*** **< 0.001**	*p* = 0.267
**Trait PA variability**	4.94 (2.1)	5.68 (2.1)	5.31 (2.1)	3.60 (1.8)	4.49 (2.3)	4.02 (2.1)	***p*** **< 0.001**	***p*** **< 0.001**	*p* = 0.726
**Trait NA**	11.51 (2.1)	11.71 (2.4)	11.61 (2.2)	12.06 (3.2)	12.94 (3.7)	12.47 (3.4)	*p* = 0.08	***p*** **= 0.004**	*p* = 0.272
**Trait NA variability**	1.37 (1.3)	1.67 (1.4)	1.52 (1.3)	1.45 (1.3)	2.04 (1.7)	1.7 (1.5)	***p*** **= 0.003**	*p* = 0.132	*p* = 0.320
**Cognitive function**									
**Pattern recognition memory (ms)**	2370.05 (560.3)	2522.03 (813.5)	2445.62 (700.0)	3109.47 (1701.9)	2947.80 (958.2)	3032.98 (1398.9)	*p* = 0.967	***p*** **< 0.001**	*p* = 0.179
**Spatial working memory (total errors)**	30.83 (18.9)	42.18 (19.7)	36.47 (20.0)	32.15 (21.0)	36.74 (23.5)	34.32 (22.2)	***p*** **< 0.001**	*p* = 0.346	*p* = 0.123
**5-Choice reaction time (ms)**	377.86 (55.3)	388.81 (61.8)	383.30 (58.7)	423.66 (101.2)	466.86 (87.6)	444.10 (97.2)	***p*** **= 0.001**	***p*** **< 0.001**	*p* = 0.053
**Match to sample visual search (ms)**	3095.51 (1010.9)	2892.37 (987.8)	2994.51 (1001.8)	4005.36 (1340.5)	4026.68 (1565.6)	4015.45 (1447.4)	***p*** = 0.488	***p*** **< 0.001**	*p* = 0.392

Significant effects are given in bold.

For cognition, females had higher errors on SWM and had longer reaction times compared to males. Age group differences emerged for PRM, 5CRT and MTS, with older adults (70-87 yrs group) performing less well on all measures, taking longer to select the correct pattern (PRM), and with slower reaction times (5CRT) and slower information processing in MTS (see Table 2).

There were social class differences for SWM and MTS (see Table 3). From post hoc tests, the main differences on these aspects of memory were between Professional and unskilled categories (*p = 0.046* and *p < 0.001*) respectively, with the professional category making fewer errors on SWM and having faster information processing times on MTS.

**Table 3:** Means (and SDs) for the state and trait measures of everyday mood and cognitive variables for social class, also showing age*social class interactions

Variable	55-70 yrs			70-87 yrs				
	Professional	Skilled	Unskilled	Professional	Skilled	Unskilled	Social class differences	Age*Social class interactions
**State mood**								
**State PA**	33.29 (5.6)	33.65 (6.3)	34.92 (6.1)	29.35 (9.2)	27.37 (9.6)	25.38 (7.4)	*p* = 0.590	*p* = 0.142
**State NA**	13.40 (4.5)	14.65 (5.4)	15.21 (5.5)	13.58 (5.5)	13.94 (5.7)	14.38 (5.1)	*p* = 0.285	*p* = 0.740
**Trait mood and mood variability measures**								
**Trait PA**	27.53 (4.8)	27.34 (6.0)	28.74 (5.6)	23.48 (8.0)	21.69 (7.5)	23.75 (6.3)	*p* = 0.235	*p* = 0.571
**Trait PA variability**	5.30 (2.2)	5.44 (2.0)	4.83 (1.8)	4.13 (2.1)	3.91 (2.0)	4.21 (2.4)	*p* = 0. 894	*p* = 0.474
**Trait NA**	11.65 (2.3)	11.59 (2.3)	11.84 (2.2)	12.16 (3.7)	12.50 (3.4)	13.28 (3.0)	*p* = 0.521	*p* = 0.677
**Trait NA variability**	1.64 (1.6)	1.44 (1.1)	1.41 (1.2)	1.63 (1.6)	1.65 (1.3)	2.42 (1.9)	*p* = 0.403	*p* = 0.194
**Cognitive function**								
**Pattern recognition memory (ms)**	2346.60 (572.9)	2500.22 (791.7)	2722.75 (734.7)	3043.21 (1269.4)	2978.45 (1509.2)	3270.22 (1223.3)	*p* = 0.375	*p* = 0.688
**Spatial working memory (total errors)**	35.56 (18.4)	34.52 (21.1)	48.57 (17.5)	29.18 (20.9)	36.63 (22.5)	38.19 (23.3)	***p*** **= 0.023**	*p* = 0.102
**5-Choice reaction time (ms)**	378.62 (53.4)	384.58 (62.9)	404.63 (61.9)	439.90 (117.9)	446.03 (91.1)	447.04 (50.7)	*p* = 0.544	*p* = 0.813
**Match to sample visual search (ms)**	3044.68 (972.9)	2882.20 (949.2)	3592.38 (1376.8)	3999.73 (1368.7)	3837,19 (1289.9)	4936,15 (2031.2)	***p*** **= 0.001**	*p* = 0.684

Significant effects are given in bold.

There were no interaction effects for either sex * age (Table 2) or age * social class (Table 3).

### Correlations between affect and cognition

Table 4 summarizes the Pearson bivariate correlations between cognition and state and trait affect and affect variability. It is worth noting that a number of small but highly significant correlations emerged. Higher state PA and trait PA were associated with fewer errors on SWM, faster reaction times and faster MTS. Higher PA variability was associated with faster reaction times and faster MTS. Higher state and trait NA were associated with poorer PRM, and higher state NA was associated with fewer errors on the SWM task.

**Table 4:** **Pearson’s bivariate correlations between state and trait measures of mood and cognitive function**

Cognitive measures	State PA	State NA	Trait PA mean	Trait PA variability (SD)	Trait NA mean	Trait NA variability (SD)
**Pattern recognition memory (ms)**	-.073	.102*	-.085	-.053	.158**	.100
**Spatial working memory (total errors)**	-.192**	-.136**	-.144**	-.090	-.063	-.031
**5-Choice reaction time (ms)**	-.198**	-.089	-.215**	-.154**	.070	.055
**Match to sample visual search (ms)**	-.176**	-.026	-.188**	-.217**	.030	-.007

*Note.* Significant correlations are given in bold, and significance levels are denoted by **p < 0.01 and *p < 0.05. Better cognitive performance is indicated by lower scores for the memory measures.

### Socio-demographic variables, state PA, trait PA and trait PA variability as predictors of cognitive function

#### Pattern recognition memory

As shown in Table 5, the socio-demographic variables (age, sex and social class) accounted for around 10% of the variance in PRM, but there was virtually no change in the *R*^*2*^ with the addition of state PA or trait PA and PA variability, which were not significant in the final model. In terms of their unique contribution to the variance in PRM (i.e., variance accounted for after controlling all other predictors in the final model), the only significant predictor was age (squared semi partial correlation, spc^2^ = 0.070) indicating that the speed of PRM is slower with increasing age (β = .289, *p* < 0.001).

**Table 5:** **Summary of hierarchical regression analyses for each of four cognitive measures as dependent variables, and socio-demographics, state and trait positive mood as predictor variables**

Measure	Step	Predictor variables	*R* ^*2*^	*∆R* ^*2*^	*F*	*p*
Pattern recognition memory (ms)						
	1	Socio-demographics	**.099**	**.099**	***F*** **(4, 359) = 9.81**	**< .001**
	2	State positive mood	.099	.000	*F*(1, 358) = 0.16	.686
	3	Trait positive Mood mean and variability	.102	.003	*F*(2, 356) = 0.51	.597
Spatial working memory – total errors						
	1	Socio-demographics	**.041**	**.041**	***F*** **(4, 359) = 3.81**	**.005**
	2	State positive mood	**.071**	**.030**	***F*** **(1, 358) = 11.69**	**.001**
	3	Trait positive Mood mean and variability	.075	.004	*F*(2, 356) = 0.69	.498
5-Choice Reaction Time (ms)						
	1	Socio-demographics	**.155**	**.155**	***F*** **(4, 359) = 16.45**	**< .001**
	2	State positive mood	.161	.006	*F*(1, 358) = 2.36	.125
	3	Trait positive Mood mean and variability	.166	.006	*F*(2, 356) = 1.19	.307
Match to sample visual search (ms)						
	1	Socio-demographics	**.158**	**.158**	***F*** **(4, 359) = 16.87**	**< .001**
	2	State positive mood	.162	.004	*F*(1, 358) = 1.52	.218
	3	Trait positive Mood mean and variability	**.178**	**.016**	***F*** **(2, 356) = 3.47**	**.032**

*Note.* Significant increases in *R*^*2*^ indicated in bold. The first step in the regression analysis involved entering socio-demographic information of age in years, sex and social class, followed by state positive mood in step two, and lastly, in step three trait measures of positive mood and its variability were entered. It should be noted that each step, after step one, included the variable(s) from the previous step(s).

#### Spatial working memory

In step one of the model, the socio-demographic variables accounted for 4% of the variance in SWM (total errors), there was a change in *R*^*2*^ with the addition of state PA in step two of the model, and no change with the addition of trait PA and PA variability in step three (see Table 5). In terms of their unique contribution to variability in SWM (total errors), the only predictor of SWM was sex (spc^2^ = 0.025), indicating that males made fewer errors than females (β = -.168, *p* = 0.003) on this test.

#### 5-choice reaction time

In step one of the model, socio-demographic variables accounted for 15% of the variance in 5CRT, with virtually no change in *R*^*2*^ with the addition of state PA and trait PA and PA variability measures in steps two and three, which were not significant in the final model (see Table 5). In terms of their unique contribution to variability in 5CRT, the only predictors were age (spc^2^ = 0.085) and sex (spc^2^ = 0.031), suggesting that age has a slowing effect on reaction times (β = .309, *p* < 0.001) and males were faster on this test compared to females (β = -.178, *p* = 0.001).

#### Match to sample visual search

As shown in Table 5, in step one of the model, socio-demographic variables accounted for 15% of the variance in MTS scores. There was virtually no change in the *R*^*2*^ with the addition of state PA, but there was an increase in step 3 of the model with the addition of trait PA and PA variability. In terms of their unique contribution to MTS mean correct latencies, the predictors of age (spc^2^ = 0.066), professional occupation (spc^2^ = 0.036), skilled occupation (spc^2^ = 0.049), and trait PA variability (spc^2^ = 0.012) were significant. In summary, being older (β = .269, *p* < 0.001) has a detrimental effect on speed of responding in this attention task. Those participants who were from professional occupations (β = -.319, *p* < 0.001) or skilled workers (β = -.364, *p* < 0.001), and those who had higher trait PA variability (β = -.118, *p* = 0.037) were faster on this task.

### Socio-demographic variables, state NA, trait NA and trait NA variability as predictors of cognitive function

#### Pattern recognition memory

As shown in Table 6, the socio-demographic variables (age, sex and social class) accounted for around 9% of the variance in PRM, but there was no change in the *R*^*2*^ with the addition of state NA in step two, nor with the addition of trait NA and trait NA variability in step three. In terms of their unique contribution to the variance in PRM, the only significant predictors were age (spc^2^ = 0.071) and having a professional occupation (spc2 = 0.010), indicating that the speed of PRM was slower with increasing age (β = .229, *p* < 0.001) and faster in those from professional occupations (β = -.178, *p* = 0.048).

**Table 6:** **Summary of hierarchical regression analyses for each of four cognitive measures as dependent variables, and socio-demographics, state and trait negative mood as predictor variables**

Measure	Step	Predictor variables	*R* ^*2*^	*∆R* ^*2*^	*F*	*p*
Pattern recognition memory (ms)						
	1	Socio-demographics	**.094**	**.094**	***F*** **(4, 367) = 9.53**	**< .001**
	2	State negative mood	.095	.001	*F*(1, 366) = 0.52	.471
	3	Trait negative Mood mean and variability	.102	.006	*F*(2, 364) = 1.31	.270
Spatial working memory – total errors						
	1	Socio-demographics	**.039**	**.039**	***F*** **(4, 367) = 3.72**	**.006**
	2	State negative mood	**.059**	**.021**	***F*** **(1, 366) = 7.98**	**.005**
	3	Trait negative Mood mean and variability	.060	.001	*F*(2, 364) = 0.04	.832
5-Choice reaction time (ms)						
	1	Socio-demographics	**.154**	**.154**	***F*** **(4, 367) = 16.67**	**< .001**
	2	State negative mood	.160	.006	*F*(1, 366) = 2.61	.107
	3	Trait negative Mood mean and variability	.163	.003	*F*(2, 364) = 0.82	.443
Match to sample visual search (ms)						
	1	Socio-demographics	**.153**	**.153**	***F*** **(4, 367) = 16.55**	**< .001**
	2	State negative mood	.153	.000	*F*(1, 366) = 0.08	.789
	3	Trait negative Mood mean and variability	.155	.002	*F*(2, 364) = 0.35	.708

*Note.* Significant increases in *R*^*2*^ indicated in bold. The first step in the regression analysis involved entering socio-demographic information of age in years, sex and social class, followed by state negative affect in step two, and lastly, in step three trait measures of negative mood and its variability were entered. It should be noted that each step, after step one, included the variable(s) from the previous step(s).

#### Spatial working memory

In step one of the model, the socio-demographic variables accounted for almost 4% of the variance in SWM (total errors); there was a change in *R*^*2*^ with the addition of state NA but no change with the addition of trait NA and trait NA variability measures (see Table 6). In terms of their unique contribution to variability in SWM (total errors), the only predictors were sex (spc^2^ = 0.019), professional occupation (spc^2^ = 0.010) and state NA (spc^2^ = 0.016) indicating that males made fewer errors than females (β = -.146 , *p* = 0.007), as did those who were from the professional occupations (β = -.184 , *p* = 0.046) and those with higher state NA (β = -.155, *p* = 0.013) on this test.

#### 5-choice reaction time

In step one of the model, socio-demographic variables accounted for 15% of the variance in 5CRT, with virtually no change in *R*^*2*^ with the addition of state NA, nor with the addition of trait NA and trait NA variability, which were not significant in the final model (see Table 6). In terms of their unique contribution to variability in 5CRT, the only predictors were age (spc^2^ = 0.109) and sex (spc^2^ = 0.027) suggesting that age has a slowing effect on reaction times (β = .338, *p* < 0.001) and males are faster on this measure compared to females (β = -.176, *p* = 0.001).

#### Match to sample visual search

As shown in Table 6, in step one of the model, socio-demographic variables accounted for 15% of the variance in MTS, with no change in *R*^*2*^ with the addition of state NA, nor with the addition of trait NA and NA variability, which were not significant in the final model. In terms of their unique contribution to MTS mean correct latencies, the predictors of age (spc^2^ = 0.109), professional occupation (spc^2^ = 0.029), and skilled occupation (spc^2^ = 0.040) were significant. In summary, being older (β = .328, *p* < 0.001) has a detrimental effect on speed of responding in this attention task. Professional (β = -.310, *p* < 0.001) and skilled workers (β = -.350, *p* < 0.001) have faster responses compared to unskilled workers on this task.

## Discussion

This study set out to determine if state and trait measures of everyday affect and its variability can predict cognitive function in healthy older adults after controlling for socio-demographic variables. Results suggested that state and trait measures of affect may have very small differential effects on cognition in older individuals. The results suggested that both state PA and NA predicted a small amount of the variance in SWM in step two of the regression models and that trait PA and its variability predicted a small amount of the variance in MTS in the final model. Bless and Fiedler (2006) claim that different affect types may have an adaptive function in relation to information processing, utilising assimilation (a person imposes an internal structure on our external world) and accommodation (the internal structure is changed as a result of external constructs). This may account for the finding that both state PA and state NA were significant predictors of SWM in step two of the models. According to this theory, during heightened PA, pre-existing attitudes and knowledge dominate information processing. Higher levels of NA promote externally focused processing, attending to external situational information which drives processing (Bless and Fiedler, 2006). This can account for both types of affect being predictive of SWM, but utilising different processes to complete the memory task. It is worth noting that only state NA was predictive of SWM on examination of the contributions of the variables in the final models. The results indicated that trait PA variability was found to result in faster information processing in MTS, and state NA was related to fewer errors on SWM.

PA variability may be related to motivation and attention which are important to performance on cognitive tasks (Forstmeier and Maercker, 2008; Hess et al. 2012), such as MTS. There may also be an underlying physiological link as elevations or changes in PA may lead to corresponding changes in arousal (Clore and Starbeck, 2006) that are associated with changes in neuromodulators in the frontal cortex, one of the areas of the brain activated during sustained attention tasks. The results support the findings of early mood induction studies that suggested fluctuations in PA were associated with changes in arousal and attention (Ashby et al. 1999). Higher levels of PA may produce more flexibility in processing and organising information, and thus enhancing cognitive performance (Isen et al. 1987). The current findings suggest that PA did not suppress processing in this sample of older adults and is in keeping with early research that suggests it might facilitate the interaction between working memory and long-term memory (Isen et al. 1987). In older people, higher PA is associated with greater motivation and engagement with the environment which, in turn, promotes increased cognitive capacity (Forstmeier and Maercker, 2008; Stine-Morrow et al. 2008). The findings of the current study are in contrast to a previous study of young adults which reported that PA was associated with reduced visual attention (Rowe et al. 2007). Recent research has implied that ageing is associated with a reduction in the intra-individual variability of PA and NA (Röcke et al. 2009). The current findings have suggested there may be small but important cognitive concomitants of this age-related change.

State NA was associated with fewer errors on SWM in the current study. Previous research suggests that NA may lead to a greater focus on the task, thereby enhancing performance (Bless and Fiedler, 2006; Clark and Isen, 1982; Schwarz, 1990). This is in contrast to a recent study monitoring daily changes in affect and cognitive function in younger adults which reported that on days where NA was elevated, performance on working memory tasks was reduced (Aoki et al. 2011; Brose et al. 2012). This reduction was related to cognitive control and motivation to perform the tasks. Some studies report no effect of NA on measures of working memory (e.g., Oaksford et al. 1996). NA in everyday life does not fluctuate to the same extent as PA, unless a stressful event occurs (Watson, 2000). The underlying biological processes of NA are thought to be different from those involved in PA (Davidson et al. 2004). Increased activation of the dopamine system, which may be related to changes in affect, may enhance processes in the prefrontal cortex (Mitchell and Phillips, 2007), the area responsible for working memory. There is a suggestion that NA is specifically related to changes in serotonin levels in the brain (Mitchell and Phillips, 2007). Drug induced increases in serotonin levels were found to have a detrimental effect on SWM (Luciano et al. 1998).

Research suggests that affect can influence cognitive performance in later life (Ashby et al. 1999), and that depression with age may increase the risk of cognitive decline and dementia (Santos et al. 2013; Singh-Manoux et al. 2010). Affective and cognitive processes share similar brain regions, identified by neuroimaging studies, such as the amygdala, orbito-frontal cortex, medial prefrontal cortex, fusiform gyrus and the inferior frontal gyrus (see Femenia et al. 2012; Forgas, 2008; Matsunaga et al. 2009; for reviews). Anatomical changes in the brain with age related to areas responsible for learning and memory may account for corresponding changes in affect and cognitive performance with age (Grady, 2000; Grady and Craik, 2000; Robbins et al. 1998; West, 1996).

The prefrontal cortex has been identified as important for the mediation of memory, cognition and emotions (Barbas, 2000; Mitchell and Phillips, 2007). Different areas of the prefrontal cortex have been implicated in different memory tasks. The caudel lateral prefrontal cortex is related to visual and auditory processing; the intraparietal and posterior cingulate are associated with attention and visual focus. Working memory is associated with activation of the thalamic multiform and parvocellular sections of the mediodorsal nucleus. The cerebellum has also been linked to the regulation of cognition and emotion (see Stoodley and Schmahmann, 2010, for a review).

The only consistent predictors of cognitive function in the current study were the socio-demographic variables of age, sex and social class which together accounted for 4-16% of the variance in the cognitive measures when entered initially into the first step of the regression analyses. There were a number of sex and age differences in relation to cognition and affect. In keeping with previous cross-sectional studies that note a decline in cognitive performance in adults over 70 years (Robbins et al. 1994; Salthouse, 2010), older individuals (>70 yrs) in this study generally performed less well on cognitive tests in comparison to the younger group. Also in keeping with previous research (Meinz and Salthouse, 1998; Robbins et al. 1994; Robbins et al. 1998), males did better on working memory tasks and had faster reaction times than females, supporting previous research. Social class differences were also observed for cognition. The finding that professional and skilled worker categories performed better than the unskilled on SWM and MTS is also in agreement with previous findings (Gallacher et al. 1999; Minicuci and Noale, 2005; Rabbitt et al. 1995; Santos et al. 2013).

There were age differences such that trait PA and trait PA variability were lower and trait NA was higher in the 70-87 years group. There were no group differences for state affect. These findings are in keeping with previous cross-sectional studies that have looked at differences in affect across age groups (Charles et al. 2001), but are in contrast to the findings of Mroczek and Kolarz (1998) who reported increased PA and decreased NA in older age groups (Birchler-Pedcross et al. 2009). That females in the current study tended to report lower PA and higher NA compared to males supports some previous findings (Crawford and Henry, 2004; Mroczek and Kolarz, 1998) but contrasts with others (Charles et al. 2001) who reported no sex differences for affect. Also females here showed greater variability in both trait PA and NA.

This study differs from previous research in that it included state and trait intra-individual variability measures of everyday PA and NA assessed over a longer duration of time compared to laboratory-based studies (Oaksford et al. 1996), or studies of affective disorders that have taken one-off measures of affect (Rabbitt et al. 1995). This enables the examination of affect variability (SD) which has not been examined extensively in previous studies of this kind. Also, CANTAB is a widely used battery of tests that has proven reliability and validity for use in older people and the tests selected are sensitive to changes in brain function with age. All the CANTAB tests were non-verbal in nature so we cannot generalise our findings to all memory domains. There is also a suggestion in the literature that cognitive function may vary from one testing time to the next, particularly in older adults, and that more than one testing session should be examined to take this variability into consideration (Brose et al. 2012; Salthouse et al. 2006).

## Conclusions

This study recruited only healthy participants with no major physical or mental health problems. For this reason, they may not be representative of typical older adults within this age group so generalisation of results is limited. Nonetheless, this paper contributes to the existing research on affect and cognition, in a natural context. It is worth noting that the contribution of state and trait PA and NA measures to cognitive function in the current study was minimal, ranging from 1-2% in the regression analyses. There were quite a few small correlations between mood and cognition (as shown in Table 4) but after controlling for the effect of socio-demographic variables, almost all of these disappeared. Socio-demographic variables accounted for between 4-16% of the variance in cognitive function. The current findings provide indirect support for some recent studies suggesting that socio-demographic factors cannot account for individual differences in cognitive function in later life (Lang et al. 2008) and that more research is needed to fully understand what other social and environmental factors are important. In order to understand more completely the relationship between affect and cognition across age, they may need to be assessed as repeated measures and, where possible, assessed at the same time.
